# Members of the majority need to actively promote diversity, equity, inclusion, and belonging

**DOI:** 10.1371/journal.pbio.3001902

**Published:** 2022-12-14

**Authors:** Cody J. Smith

**Affiliations:** Department of Biological Sciences, University of Notre Dame, Notre Dame, Indiana, United States of America

## Abstract

Interesting in including diversity, equity, inclusion and belonging in your lab but not sure how to start? This Perspective speaks to everyone, including those in the "majority", about how to get involved.

## Teaser

The responsibility for promoting diversity, equity, inclusion, and belonging (DEIB) too often falls on scientists from minority groups. Here, I provide a list of potential strategies that members of the majority can easily do to step up and get involved in DEIB.

When diversity, equity, inclusion, and belonging (DEIB) is achieved, science and society will be better for everyone [1–3], yet much of the effort to make science more inclusive has been driven by those from minority groups. The effort needed to induce change in scientific culture should not be the responsibility of those who are burdened by it. I urge those of us in the “majority” to get involved in making the changes needed for everyone in science to excel. Below, I outline 10 actions, based on suggestions from the literature [2,4–8] and from my own experiences, that those of us in the majority can do to improve DEIB in science.

**Fig 1:**
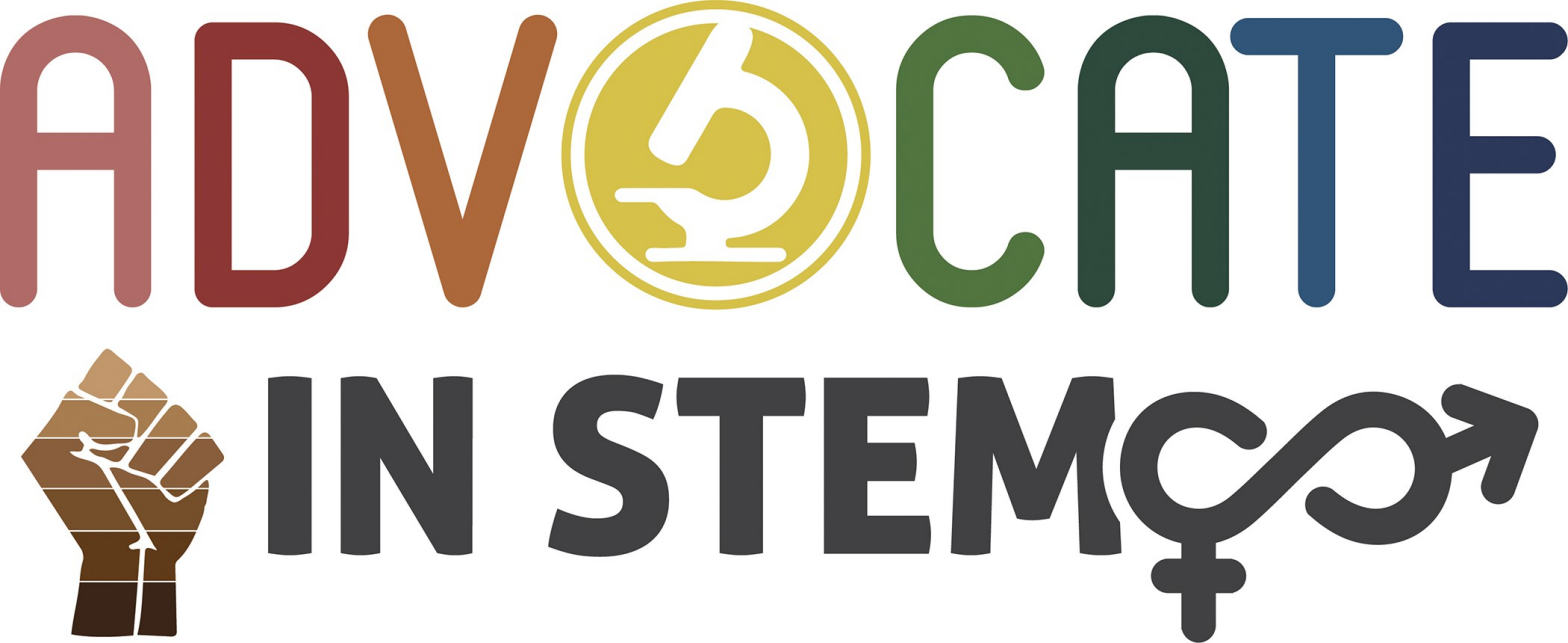
Advocate in STEM. A drawing that can be displayed on all presentations and lab spaces, indicating a lab’s goal to advocate for all individuals.

1. Listen to people’s experiences. As a white male, I had little understanding of the systemic barriers that exist in science. While many search for the overt act of racism or sexism as an example, barriers can exist in more secretive ways [2,9–11]. Reading the current and growing literature by people from underrepresented groups can provide valuable perspectives from others [2,9–12]. If your colleagues are willing to share their experiences, ask questions like: What have you experienced that revealed science could be more inclusive? What have you seen that has increased inclusiveness in science? And, what can I do, as someone in the majority, to help make science more inclusive?
2. Check your implicit biases. Implicit bias is rampant in most aspects of science, including speaker selection, awards, publications, and promotions [2,9,10]. If you want to make change, you first should realize your own implicit biases and work to remove them [2,5,8]. Project implicit has produced some tests to reveal implicit biases.
3. Stop interrupting. Take some time to watch the dynamics of meetings. What you are likely to realize is that the majority of people who interrupt others in a meeting are white men. Once I became aware of this fault, I was embarrassed by my behavior. Please let your colleagues finish their thought before interrupting. If you identify someone overly interrupting a colleague, ask them to let that person finish and invite the person who was speaking first to finish their point. If your excitement ever prevents you from not interrupting, acknowledge what you did and apologize, preferably publicly and in the same meeting so that others can learn.
4. Advocate like you do science. In research, we consistently learn from our efforts until we solve an issue. As you take a lead to impact DEIB, you will similarly experience mistakes. It is helpful to approach DEIB efforts much like we do science, constantly learning and adapting with the hope that a solution will eventually be found.
5. Advocate for compensation. It is not a secret that individuals in science from underrepresented groups, including women [2,4], persons of color [2,4], LGBTQ individuals, first-generation scholars, those from marginalized indigenous groups, and other individuals underrepresented in science are burdened with extensive service [2,4,13]. Such individuals are also the ones that trainees from underrepresented groups seek for mentorship [4]. Those of us from majority groups need to advocate for compensation for those efforts [4–6]. Such efforts must be included in tenure and promotion [5,6]. Universities could also provide an administrative supplement similar to that provided to Directors who have additional responsibilities beyond being a professor [4]. It is our responsibility to push the school’s leaders to compensate individuals for the workload that many inadvertently sign up for or are forced into doing for “the greater good.” We need to advocate that service be distributed fairly, including assigning scholars from majority groups to DEIB tasks [5,6].
6. Recognize that patience is a luxury of the majority. Those in the majority who are not overly burdened by DEIB barriers have the privilege to wait for change [4]. Unfortunately, that is not the reality for those in the minority. Try addressing this by brainstorming solutions for both the immediate and distant future [3]. For example, my lab has taken a long approach to improving representation in academia by working with a pre-school to demonstrate to children that there are scientists that look like them, hopefully before biases are introduced. But it is important to couple such initiatives with ones that will impact DEIB sooner, like leading DEIB discussions at campus invites [14].
7. Get in the room. One of the major frustrations about DEIB is the lack of participation in DEIB events. If your colleague organizes a DEIB event, make an effort to attend [2]. While you may not feel like you can contribute to the discussion, you will likely learn a great deal from listening [14]. Beyond the support of your colleagues, your attendance demonstrates your priorities to your trainees. Additionally, encourage other colleagues to attend, explain to them that this is an important part of improving science and making your diverse community colleagues and trainees feel appreciated [14]. If you have the privilege to schedule non-DEIB events, be careful they do not conflict with DEIB events.
8. Train others to advocate. Developing a detailed training plan to teach DEIB advocacy is an excellent opportunity to make an impact [7]. For example, scientists in my lab take implicit bias tests and read “The Autobiography of a Transgender Scientist,” exercises that reveal the biases in science [12]. Like others, we have slack channels dedicated to advocacy. Reserving at least 1 lab meeting a year to only discuss DEIB is also helpful. For my lab, this led us to produce a logo to display in presentations and on lab doors to show that the lab is a safe place where everyone can be advocated for (Fig 1). With experience in DEIB efforts, students become more likely to carry on the cause.
9. Include DEIB in the classroom. It is important to consider representation in your classroom material [6]. During a self-assessment of my syllabus, I recognized a missed opportunity to emphasize DEIB. I subsequently changed all my primary readings in the syllabus to be from authors who are women, from underrepresented groups, or advocates of women. In lectures, I emphasize the author’s discoveries while simultaneously introducing the authors to the class. In doing this, students can leave your classroom with an understanding of the barriers that still exist in science, blossoming more students that champion DEIB.
10. Listen to individuals like Ben Barres, who was the first openly transgender member of the National Academy of Sciences and a tireless advocate for DEIB. In his communications, he shared his unique perspective of experiencing science as both a woman and a man [12]. I would encourage you to read Ben’s autobiography [12] or listen to talks when Ben famously halted discussion of science to make comments regarding DEIB, particularly sexual harassment. Ben’s impact is best exemplified by the number of women leading the field of glial biology. Many of these women trained with Ben or were advocated for by him. Ben’s efforts showed how one person can make a significant impact in DEIB.

As you get involved, it is important to lean into any discomfort you may experience and to really listen to those around you. DEIB work is highly passionate and each person experiences it from a different perspective. We should assume good intentions when people undertake DEIB efforts and appreciate that there are many approaches that can improve DEIB in academia. Like any challenging scientific question, DEIB will be solved by a collection of people pursuing change from different perspectives and working together to find the best approaches and solutions.
